# The National Pension Fund

**Published:** 1887-10-08

**Authors:** Sidney J. Browne

**Affiliations:** Cottonera Station Hospital, Malta


### THE NATIONAL PENSION FUND.

IT will save a good deal of trouble if nurses and other hospital officials who propose joining the National Pension Fund will send their names and other particulars to *the Secretary of The Hospitals Association*, Norfolk House, Norfolk Street, W.C., and not to the Editor of THE HOSPITAL. Names continue to arrive in considerable numbers, but if those interested desire the Fund to be started before the end of the Jubilee year they must influence and interest all their friends immediately. Readers will please note that in sending in their names, nurses and other hospital officials are not committing themselves to join any particular Fund, but simply stating their willingness to join "a Pension Fund if the rules and regulations are such as they approve." One thousand and fifty names have been received to date, including ninety from St. John's House.

NAMES FROM MALTA.

I BEG to enclose my name and the names of two other sisters, who are working with me, as members of the National Pension Fund. If we remain in the service until we are sixty, we are entitled to a small Government pension, but as it is a very small amount, not sufficient to support us in our old age, we should think it a great boon to be allowed to join the National Pension Fund.

SIDNEY J. BROWNE.

Cottonera Station Hospital, Malta.
Sept. 26th, 1887.

